# Threat or strength? Study on the difference of the degree of aging on the efficiency of medical resources utilization in China

**DOI:** 10.3389/fpubh.2026.1716055

**Published:** 2026-03-20

**Authors:** Juan Luo, Ke-ting Xia, Chi-yue Huang

**Affiliations:** 1 School of Management, Shanghai University of Engineering Science, Shanghai, China; 2 Shanghai University of Engineering Science, Shanghai, China

**Keywords:** healthcare resources, Malmquist index, population aging, three-stage data envelopment analysis (DEA) model, utilization efficiency

## Abstract

**Introduction:**

Under the dual pressures of accelerating population aging and the increasing burden of chronic diseases among the older adults in China, the contradiction between surging healthcare demand and resource supply has become increasingly pronounced. The efficiency of healthcare resource utilization has thus emerged as a critical issue for optimizing resource allocation.

**Methods:**

This study employs a three-stage Data Envelopment Analysis (DEA) model and the Malmquist index to conduct static and dynamic analyses of healthcare resource efficiency across China's 31 provinces from 2010 to 2022. Regions are categorized into mild, moderate, and severe aging based on their aging intensity.

**Results:**

Findings indicate that aging severity is not the core determinant of healthcare efficiency; rather, resource allocation structures and internal management standards are pivotal.

**Discussion:**

National efficiency exhibits fluctuating, mild decline, with inefficient technological progress being the primary constraint on total factor productivity growth. Scale inefficiencies represent a structural bottleneck in enhancing healthcare system efficiency, particularly concerning resource allocation and demand matching. Furthermore, environmental factors exert a significant influence on efficiency, with regional efficiency generally improving after their exclusion. Consequently, the study recommends implementing differentiated regional resource allocation strategies, promoting smart healthcare service models, and optimizing external policies and economic environments to systematically enhance the utilization efficiency of China's healthcare resources and proactively address the long-term challenges posed by population aging.

## 1 Introduction

The wave of population aging is sweeping across the globe at an unprecedented scale and pace, emerging as one of the most influential social transformations of our era. China hosts the world's largest older adult's population, with its aging process accelerating continuously. According to National Bureau of Statistics data, by the end of 2024, the population aged 65 and above reached 220 million, accounting for 15.6% of the total population; projected to rise to 24.2% by 2035 and 32.6% by 2050 (1). The rigid demand for healthcare resources from these vast older adults' cohort is increasingly evident, with the complexity and diversity of their health issues posing unprecedented challenges to the healthcare security system. According to a World Economic Forum survey, the prevalence of chronic diseases among Chinese individuals aged 60 and above exceeds 75%, while the number of older adults people with full or partial disability nationwide has surpassed 44 million. The treatment and management of these conditions necessitate sustained, long-term investment in medical resources, encompassing pharmaceuticals, rehabilitation care, and specialized medical services. Concurrently, older adults exhibit increasingly diverse healthcare needs, extending beyond routine medical consultations to include rehabilitation care, end-of-life support, and mental health assistance. Consequently, traditional healthcare service models require adaptation and enhancement to accommodate these evolving needs.

Despite increasing investment in healthcare in recent years, the growth rate of medical resources has failed to keep pace with the rising demand driven by population aging and shifts in disease patterns. Shortfalls persist in the number of hospital beds, healthcare personnel, and medical equipment within healthcare institutions. According to the 2024 Statistical Bulletin on China's Health Development, by the end of 2024, healthcare institutions provided 10.37 million beds, equating to 7.23 beds per thousand inhabitants. The number of licensed (assistant) physicians per thousand inhabitants stood at 3.40, while the number of registered nurses reached 4.16 per thousand inhabitants by the end of 2023. Nevertheless, significant gaps persist when compared to developed nations. Concurrently, some healthcare institutions suffer from poor management and inefficient processes, leading to waste and underutilization of medical resources. Moreover, inefficient coordination between healthcare institutions at different levels, coupled with inadequate referral mechanisms and delayed information sharing, further exacerbates the waste and inefficient utilization of medical resources. This has made the imbalance between supply and demand for healthcare resources, particularly against the backdrop of an aging population, a pressing social issue requiring urgent resolution. Consequently, how to rationally utilize existing medical resources and alleviate this supply-demand contradiction represents a critical practical challenge that demands immediate attention.

## 2 Literature review

The academic community generally defines healthcare resources as the collective term for the production factors that deliver medical services, encompassing medical equipment, healthcare personnel, pharmaceuticals, and so forth. This definition enjoys broad acceptance among scholars, with extensive research on healthcare resources conducted by academics both domestically and internationally. In recent years, studies concerning the efficiency of health insurance, the allocation of healthcare resources, and the efficiency of medical services have become focal points within the academic sphere.

Regional disparities and dynamic evolution in healthcare resource allocation efficiency remain a research focus. Yaxin et al. (2) employed a three-stage Data Envelopment Analysis (DEA) model to assess the efficiency of healthcare resource allocation across China's 31 provinces from 2005 to 2019. Their findings indicate that regional differences primarily stem from inter-regional variations, exhibiting positive spatial autocorrelation that progressively intensifies. Meiling et al. (3) revealed that while China's healthcare resource efficiency has generally improved, effective allocation remains elusive. Eastern regions exhibit optimal efficiency, with shortages of medical equipment and personnel being key constraints. Western regions experience greater efficiency fluctuations due to policy and environmental influences. Peng et al. (4) observed that HRDI values for various healthcare resource indicators decrease progressively from east to west across regions. Shengen et al. (5) concluded that megacities generally demonstrate poor allocation and utilization efficiency of healthcare resources, alongside redundant investment. Xintong et al. (6) contend that departmental staffing in medical institutions fails to meet developmental demands. Research findings on evaluating healthcare service efficiency and its influencing factors are abundant. Rui et al. (7) employed the DEA-Tobit model to analyze the efficiency of rural primary healthcare services in western regions from 2017 to 2019, identifying low pure technical efficiency as the primary cause of overall inefficiency, with per capita disposable income and population density among rural residents being key influencing factors. Guoyuan et al. (8) demonstrated that eastern regions exhibit superior efficiency compared to central and western regions, with provincial disparities showing no significant convergence and instead exhibiting an expanding trend. Regis-Hernández et al. (9) drawing from a real-world case study in Montreal, Canada, concluded that distributive equity in healthcare resource allocation impacts daily operations and consequently affects resource utilization efficiency; Chen et al. (10) concluded that the scale of healthcare human resources promotes equity in medical services but inhibits service efficiency. Liang Binghua and Zhai Shaoguo employed LSDVC analysis to identify population density and population aging as significant factors affecting healthcare resource utilization levels (11). Jinlong et al. (12) after controlling environmental factors, observed that per capita GDP and population density exerted predominantly positive effects on adjusted healthcare resource efficiency values. Fang B and Li M's calculations reveal that total factor productivity in China's healthcare institutions is declining. Resource allocation inefficiencies primarily stem from redundant institutional numbers and lax outpatient services. Regional GDP levels exert a significant positive influence on efficiency improvements (13). This finding corroborates Zhai Jinlong et al.'s conclusion regarding the positive effect of per capita GDP on efficiency. Ouyang Le demonstrated that population aging impacts healthcare resource allocation efficiency, necessitating increased investment and structural adjustments to meet older adults populations' healthcare needs, thereby enhancing resource allocation efficiency (14). Xing et al. (15) analysis of 14 institutions within the Qiqihar Medical Group also underscores that institutional planning must comprehensively consider resource distribution and demographic characteristics. Progress has also been made in efficient research within specialized contexts. Zhou Zichao, against the backdrop of the COVID-19 pandemic, employed a DEA-Tobit two-stage framework to assess provincial healthcare expenditure efficiency from 2008 to 2018. Findings indicate efficiency is constrained by technological innovation rates, negatively impacted by fiscal decentralization, while positively influenced by healthcare policies and aging levels (16). Yao and Jiacheng (17) employed entropy analysis, a global non-expected output SBM model, and a VAR model to measure provincial digital development levels and healthcare resource allocation efficiency during the study period, while examining the dynamic relationship between the two.

Given that research into healthcare resource utilization spans multiple disciplines and levels, the classification is not absolute, and overlap may occur between different studies. Moreover, most studies on healthcare resources in China focus on regional disparities between eastern, central and western regions, or examine the impact of aging rates on healthcare resources within specific areas, with few studies categorizing regions based on aging levels. However, as aging intensifies, there is an urgent need to investigate healthcare resource utilization and its influencing factors across regions with differing aging profiles. This research aims to provide insights for enhancing the efficiency of healthcare resource utilization in future aging societies.

## 3 Data and methods

### 3.1 Data sources

This paper collates and analyses data on the allocation and utilization of healthcare resources across China's 31 provinces, municipalities and autonomous regions from 2010 to 2022. Statistical information is sourced from the China Health Statistics Yearbook (2011-2023), China Statistical Yearbook, and China Population and Employment Yearbook.

### 3.2 Research methodology

This paper employs a three-stage DEA model to eliminate external environmental factors (18). Utilizing relevant data from 31 provincial regions across China spanning 2010–2022, it analyses the efficiency of healthcare resource utilization against the backdrop of population aging. Building upon this, it constructs a Malmquist productivity index that accounts for external environmental factors and random errors, thereby dynamically examining the developmental trends in China's public health resource allocation.

#### 3.2.1 Three-stage DEA model

The first phase of this study employs a traditional DEA model, selecting an input-oriented BCC model to measure the efficiency of healthcare resource allocation across China's 31 provinces. This assessment accounts for the influence of external environmental factors and statistical noise (19), In the BCC model, the calculation of efficiency values essentially involves solving a linear programming problem. By optimizing the weight coefficients of inputs and outputs, it maximizes the efficiency value of the decision unit under given constraints. There are N decision units, where N=31. Each decision unit utilizes m types of medical resource inputs to generate s types of medical service outputs, as described by the following formula:


$$\begin{array}{l} \underset{u_{r},v_{i},u_{0}}{Max\;\theta =}\overset{s}{\underset{r=1}{\sum }}u_{r}y_{rj_{0}}-u_{0}\\ s.t.\overset{m}{\underset{i=1}{\sum }}v_{i}x_{ij_{0}}=1,\\ \overset{s}{\underset{r=1}{\sum }}u_{r}y_{rj}-\overset{m}{\underset{i=1}{\sum }}v_{i}x_{ij}-u_{0}\leq 0\left(j=1,2,\cdots \;,N\right)\\ u_{r},v_{i}\geq 0 \end{array}$$

For the j_0_th decision-making unit (DMU), its efficiency value θ is obtained by solving the following linear programming problem. The decision variables in this programming are the output weight coefficients u_r_ (r = 1,2,…,s), the input weight coefficients v_i_ (I = 1,…,m), and the free intercept term u_0_ representing returns to scale. The efficiency value θ is the objective function value obtained by optimizing these weight coefficients. y_rj_ denotes the rth output of the jth DMU, x_ij_ represents the ith input of the jth DMU, u_r_ and v_r_ are the output and input weight coefficients, respectively, and u0 is the scale-of-return adjustment term.

In the second stage: The efficiency scores calculated in the first stage of DEA incorporate the combined effects of environmental factors, managerial inefficiency, and random noise. To isolate these disturbances, this study employs Stochastic Frontier Analysis (SFA) to regress and adjust the slack variables from the first stage. For each input indicator, we construct the following SFA regression equation:


$$\begin{array}{l} S_{ij}=\beta_{0}+f\left(Z_{j};\beta_{i}\right)+v_{ij}+\mu_{ij};i=1,2,\cdots \;,m;j=1,2,\cdots \;,N \end{array}$$

Where S_ij_ denotes the slack value for input i in decision unit j; Z_j_ is a vector of observable external environmental variables, specifically including the medical subsidy revenue, urbanization rate, old-age dependency ratio, and per capita regional GDP of the j-th region. These variables represent exogenous factors beyond the control of decision-making units in the short term. β0 is the intercept term, representing the system's inherent average relaxation level after controlling for all observable environmental variables. with β_i_ being its coefficient; ν_ij_ +μ_ij_ constitutes the mixed error term, with ν_ij_ signifying random disturbance and μ_ij_ indicating managerial inefficiency.

Through SFA regression, we can not only determine the direction and magnitude of environmental variables' impact on slack values, but more importantly, we can isolate managerial inefficiency u_ij_ and random error v_ij_. Secondly, based on the SFA regression function, an SFA model formula is constructed to assess the efficiency of China's healthcare resource utilization after controlling for external environmental factors and excluding statistical noise from the inspection process. The formula is as follows:


$$\begin{array}{r} X_{ij}^{A}=X_{ij}+\left[\max\left(f\left(Z_{i};{\hat{\beta }}_{j}\right)\right)-f\left(Z_{j};{\hat{\beta }}_{i}\right)\right]\\ +\left[\max\left(v_{ij}\right)-v_{ij}\right];i=1,2,\cdots \;,m;j=1,2,\cdots \;,N \end{array}$$

Among these, $X_{ij}^{A}$ represents adjusted inputs; X_ij_ denotes unadjusted inputs;$\left[\max\left(f\left(Z_{j};{\hat{\beta }}_{i}\right)\right)-f\left(Z_{j};{\hat{\beta }}_{i}\right)\right]$ indicates adjustments for external environmental factors;[max(*v*_*ij*_)−*v*_*ij*_] signifies normalizing all decision units to the same level.

In the third stage, the adjusted DEA model employs the modified input variables alongside the initial output variables to recalculate the efficiency of each decision unit. The efficiency values thus computed constitute the final outcomes after controlling for external environmental factors.

#### 3.2.2 Malmquist index method?

The Malmquist index method is a dynamic productivity measurement technique proposed by Swedish economist Sten Malmquist in 1953. Its core principle involves constructing productivity indices through distance function ratios, thereby overcoming the limitations of traditional static efficiency assessments. It is primarily employed to analyze productivity changes between distinct periods.

At period t, given the output level y^t^, the input distance function A for the kth decision-making unit (DMU) is defined as the ratio of the actual input vector $D^{t}(x_{k}^{t},y_{k}^{t})$ to the minimum input vector on the production frontier. $x_{k}^{t}$ higher value indicates production is closer to the frontier; if $D^{t}(x_{k}^{t},y_{k}^{t})$, it signifies the DMU has achieved technical efficiency at period t.

The distance function can be solved through DEA model linear programming. Using the technology of period t as the reference, the distance function value for DMU k can be obtained by solving the following linear programming:


$$\begin{array}{l} \;{\left[D^{t}\left(x_{k}^{t},y_{k}^{t}\right)\right]}^{-1}=\min\;\theta \\ s.t.\overset{N}{\underset{j=1}{\sum }}\lambda_{j}x_{ij}^{t}\leq \theta x_{ik}^{t},i=1,\ldots ,r\\ \;\overset{N}{\underset{j=1}{\sum }}\lambda_{j}y_{rj}^{t}\geq y_{rk}^{t},r=1,\ldots ,s\\ \;\lambda_{j}\geq 0,j=1,\ldots ,N \end{array}$$

Where θ represents the technical efficiency value, λ_j_ denotes the weight coefficient, N indicates the number of decision-making units, and m and s represent the number of input and output indicators, respectively.

Based on the distance function derived from Data Envelopment Analysis (DEA), the formula for calculating the Malmquist index is:


$$\begin{array}{l} M\left(x^{t+1},y^{t+1},x^{t},y^{t}\right)={\left[\frac{D^{t}\left(x^{t+1},y^{t+1}\right)}{D^{t}\left(x^{t},y^{t}\right)}\times \frac{D^{t+1}\left(x^{t+1},y^{t+1}\right)}{D^{t+1}\left(x^{t},y^{t}\right)}\right]}^{1/2} \end{array}$$

*D*^*t*^(*x*^*t*+1^, *y*^*t*+1^): Distance function between inputs and outputs in period t+1 based on period t technology; *D*^*t*^(*x*^*t*^, *y*^*t*^): Distance function between inputs and outputs in period t, benchmarked against period t+1 technology; *D*^*t*+1^(*x*^*t*+1^, *y*^*t*+1^): Distance function between inputs and outputs in period t+1, benchmarked against period t+1 technology; *D*^*t*+1^(*x*^*t*^, *y*^*t*^): Distance function between inputs and outputs in period t, benchmarked against period t+1 technology. If M > 1, productivity has increased; If M = 1, productivity remains unchanged; If M < 1, productivity has decreased.

The Malmquist productivity index can be further decomposed into the efficiency change index (EC) and the technological change index (TC), expressed as:


$$\begin{array}{l} M\left(x^{t+1},y^{t+1},x^{t},y^{t}\right)=EC\times TC\\ \text{where }EC=\frac{D^{t+1}\left(x^{t+1},y^{t+1}\right)}{D^{t}\left(x^{t},y^{t}\right)}\\ TC={\left[\frac{D^{t}\left(x^{t+1},y^{t+1}\right)}{D^{t+1}\left(x^{t+1},y^{t+1}\right)}\times \frac{D^{t}\left(x^{t},y^{t}\right)}{D^{t+1}\left(x^{t},y^{t}\right)}\right]}^{1/2}; \end{array}$$

Under the assumption of variable returns to scale, the efficiency change (EC) can be further decomposed into pure technical efficiency change (PTC) and scale efficiency change (SC): EC = PTC × SC,


$$\begin{array}{r} \text{where }PTC=\frac{D_{VRS}^{t+1}(x^{t+1},y^{t+1})}{D_{VRS}^{t}(x^{t},y^{t})}\\ SC=\frac{D_{CRS}^{t+1}(x^{t+1},y^{t+1})/D_{VRS}^{t+1}(x^{t+1},y^{t+1})}{D_{CRS}^{t}(x^{t},y^{t})/D_{VRS}^{t}(x^{t},y^{t});} \end{array}$$

In this equation, D_CRS_ and D_VRS_ represent the distance functions under assumptions of constant returns to scale and variable returns to scale, respectively. Through this decomposition, the sources of changes in healthcare resource utilization efficiency can be more clearly identified: if PTC > 1, it indicates improved management; if SC > 1, it signifies optimized scale structure; and if TC > 1, it reflects technological progress driving the production frontier upward.

### 3.3 Construction of Indicator System

This study builds upon prior literature, integrating input-output theory, Pareto optimality theory, and public administration theory. Based on a three-dimensional influence framework encompassing ‘input-output-environment,' relevant indicators were selected. Data from the China Health Statistics Yearbook were utilized: the number of healthcare professionals per thousand population, the number of hospital beds per thousand population, and the number of healthcare institutions were chosen as indicators of healthcare resource input. The following indicators from the China Health Statistics Yearbook were selected as medical resource output indicators: outpatient service consultations, hospital inpatient admissions, and bed turnover rate^1^. Traditional DEA models assume all decision-making units operate under identical environmental conditions. However, external environments vary across units, with these factors influencing input-output performance and consequently affecting efficiency evaluation outcomes. Therefore, this study selects urbanization rate, per capita regional GDP, old-age dependency ratio, and medical fiscal subsidy income as external environmental variables representing economic development levels, demographic factors, and government policy influences respectively. These indicators, combined with input-output metrics, collectively form the three-stage DEA model indicator system, as detailed in Table 1.

**Table 1:** Three–stage DEA model indicators.

**Indicator type**	**Indicator**	**Unit**
Input Indicator (I)	Number of healthcare professionals per thousand population (I_1_)	Persons per thousand
	Number of healthcare institution beds per thousand population (I_2_)	Beds per thousand persons
	Number of healthcare institutions (I_3_)	Units
Output indicators (O)	Number of outpatient consultations at hospitals (O_1_)	Persons
	Hospital inpatient admissions (O_2_)	Individuals
	Bed Turnover Rate(O_3_)	%
Environmental Variables (E)	Medical Financial Subsidy Income (E_1_ )	Ten thousand yuan
	Urbanization rate (E_2_)	%
	Old–age dependency ratio (E_3_)	%
	Per capita regional GDP (E_4_)	Ten thousand yuan

## 4 Analysis of measured efficiency of China's medical resources utilization in the context of population aging

### 4.1 Static efficiency analysis

According to the National Bureau of Statistics standard^2^, China's 31 provinces are categorized into mildly, moderately, and severely aged regions according to their aging levels, as shown in Table 2.

**Table 2:** Classification of aging regions.

**Degree of aging**	**Region**
Mild aging	Fujian Province, Gansu Province, Guangdong Province, Guangxi Province, Guizhou Province, Hainan Province, Jiangxi Province, Ningxia, Qinghai Province, Tibet, Xinjiang and Yunnan Province.
Moderate aging	Anhui Province, Beijing, Hebei Province, Henan Province, Heilongjiang Province, Hubei Province, Hunan Province, Jilin Province, Jiangsu Province, Inner Mongolia, Shandong Province, Shanxi Province, Shaanxi Province, Sichuan Province, Tianjin Municipality, Zhejiang Province and Chongqing Municipality.
Heavily aging	Shanghai Municipality, Liaoning Province

Multicollinearity tests serve as a crucial prerequisite for assessing the validity of model specifications and the robustness of results. Table 3 presents the multicollinearity test results for the data indicators of the three-stage DEA model.

**Table 3:** Multicollinearity results.

**Variable**	**VIF**	**1/VIF**
Per capita regional GDP	4.94	0.1683
Health professionals per thousand population	4.73	0.211222
Number of healthcare institution beds per thousand population	3.56	0.280941
Urbanization rate	3.47	0.288357
Old–age dependency ratio	2.79	0.358906
Medical subsidy income	2.53	0.395665
Number of healthcare institutions	1.86	0.538574
Mean VIF	3.41	

Based on the results of the multicollinearity test, the variance inflation factor (VIF) for all variables was less than 5 (mean 3.41), indicating no severe multicollinearity issues among the variables. Among these, per capita regional GDP (VIF = 4.94) and healthcare professionals per thousand population (VIF = 4.73) exhibited relatively higher correlations, yet remained within acceptable limits, reflecting the intrinsic linkage between economic development and healthcare resource allocation. All other variables displayed VIF values below 3.5, indicating good independence. Overall, the current variable set is suitable for three-stage DEA analysis, yielding results with sound stability and reliability.

To validate the reliability and generalizability of the research conclusions, ensuring efficiency assessment outcomes remain fundamentally unchanged by minor variations in model specification, indicator selection, or data processing methods, robust tests were conducted prior to the three-stage DEA model analysis. To ensure the reliability and universality of the research conclusions, and to verify that the efficiency evaluation results will not undergo fundamental changes due to model settings, indicator selection, or sample fluctuations, this paper employs the Bootstrap resampling method for robustness testing. Specifically, Bootstrap resampling was applied to the coefficient estimates of each input variable and environmental variable in the three output equations reported in Table 4 to assess the robustness and statistical significance of these coefficient estimates. The Bootstrap method constructs empirical distributions by repeatedly sampling with replacement from the original sample, thereby estimating sampling variables and confidence intervals for statistical measures. Given the panel structure of the data, a province-level (DMU-level) clustered Bootstrap was employed. The Bootstrap repetition count was set to 500, sufficient to yield stable standard error estimates and confidence intervals. The results are presented in Table 4.

**Table 4:** Bootstrap robustness test results.

**Variable**	**Bed turnover rate**	**Hospital inpatient services number of admissions**	**Number of outpatient consultations**
Health technicians per 1,000 population	−7.709^***^	−392,650^***^	−5.221e+06^***^
	(2.314)	(142,594)	(1.985 × 106)
Number of healthcare Institution beds per thousand population	−0.0965^*^	772,721^***^	2.585 × 106
	(2.555)	(155,707)	(2.370e+06)
Number of healthcare institutions	0.00056^***^	117.4^***^	1,917^***^
	(0.00019)	(10.65)	(352.5)
Per capita regional gross domestic product	−8.76e−05	3.643	30.07
	(0.000175)	(7.087)	(144.7)
Old–age dependency ratio	−1.572^**^	−14,856	1.150e+06^**^
	(0.673)	(26,649)	(480,170)
Financial subsidy income	−5.46 × 10^−6***^	0.381^***^	5.275^*^
	(1.57 × 10^−6^)	(0.104)	(2.882)
Urbanization rate	21.68	7.802 × 10^***^	2.493 × 108^***^
	(69.39)	(1.441 × 106)	(3.717 × 107)
Sigma_u	32.46^***^	1.497 × 106^***^	4.961 × 107^***^
	(4.570)	(84,212)	(2.387 × 106)
Sigma_e	17.14^***^	806,409^***^	1.356 × 107^***^
	(1.199)	(40,391)	(813,285)
Constant	331.1^***^	−5.257e+06^***^	−1.139 × 108^***^
	(27.96)	(731,668)	(1.933 × 107)
Observations	403	403	403
Number of regions	31	31	31
Log likelihood	−1777	−6112	−7270

Standard errors in parentheses ^***^p < 0.01, ^**^p < 0.05, ^*^p < 0.1.

According to Bootstrap robustness tests, the direction and significance of each environmental variable's impact on healthcare resource redundancy remained stable. Notably, the number of healthcare professionals per thousand population, the number of healthcare institutions, and fiscal subsidy income exerted highly significant and consistently positive effects on both bed turnover rates and inpatient admission redundancy, confirming the pivotal role of healthcare human resources and institutional scale. The positive and significant impact of the old-age dependency ratio on outpatient service redundancy is also confirmed, reflecting the pressure of an aging population on outpatient demand. Furthermore, the significant positive effect of urbanization rate on both inpatient and outpatient service redundancy remains robust, indicating that the urbanization process continues to exacerbate the strain on healthcare resources. All core conclusions remain fundamentally unchanged across different Bootstrap samples, demonstrating that the estimation results of the three-stage DEA model possess high reliability and statistical robustness.

#### 4.1.1 First stage DEA analysis

Using DEAP 2.1 as the operational environment, the collected data was first applied to an input-based BCC model (20). Raw input and output data were input into the DEAP 2.1 software to calculate comprehensive technical efficiency, pure technical efficiency, scale efficiency, and slack values for each input across all decision units from 2010 to 2022. Taking 2022 as an example, the results are presented in Table 5.

**Table 5:** Average efficiency of healthcare resource utilization and its component efficiency across regions with different levels of aging.

**Level of aging**	**Region**	**Initial values in phase I** ^**a**^				**Adjusted values in stage three** ^**b**^			
		**Overall efficiency**	**Pure technical efficiency**	**Scale efficiency**	**Rewards for scale**	**Overall efficiency**	**Pure technical efficiency**	**Scale efficiency**	**Scale returns**
Mild aging	Jiangxi	0.906	1	0.906	irs	0.906	1	0.906	irs
	Ningxia	0.765	1	0.765	irs	0.851	1	0.851	irs
	Fujian	0.792	1	0.792	irs	0.792	1	0.792	irs
	Guangdong	1	1	1	–	1	1	1	–
	Tibet	0.477	1	0.477	irs	0.487	1	0.487	irs
	Guangxi	0.848	0.932	0.909	irs	0.879	0.969	0.907	irs
	Yunnan	0.896	0.968	0.926	irs	0.980	1	0.980	irs
	Xinjiang	0.697	0.968	0.720	irs	0.728	0.963	0.756	irs
	Guizhou	0.785	0.925	0.849	irs	0.849	0.933	0.909	irs
	Hainan	0.600	0.991	0.605	irs	0.760	1	0.760	irs
	Gansu	0.604	0.934	0.646	irs	0.677	0.983	0.688	irs
	Qinghai	0.519	0.910	0.570	irs	0.691	0.951	0.727	irs
Mean	0.741	0.969	0.764		0.800	0.983	0.814	
Moderate aging	Hubei	0.916	0.975	0.940	irs	0.954	0.988	0.965	irs
	Jiangsu	1	1	1	–	1	1	1	–
	Tianjin	0.659	1	0.659	irs	0.685	1	0.685	irs
	Zhejiang	1	1	1	–	1	1	1	–
	Chongqing	0.822	0.992	0.828	irs	0.874	1	0.874	irs
	Anhui	0.881	1	0.881	irs	0.965	1	0.965	irs
	Henan	0.913	1	0.913	drs	0.913	1	0.913	drs
	Sichuan	0.942	1	0.942	drs	0.942	1	0.942	drs
	Hunan	0.870	0.909	0.958	irs	0.870	0.911	0.955	irs
	Beijing	0.825	0.965	0.855	irs	0.816	0.932	0.876	irs
	Hebei	0.741	0.901	0.822	irs	0.741	0.907	0.817	irs
	Shandong	0.864	1	0.864	drs	0.864	1	0.864	drs
	Shaanxi	0.674	0.807	0.835	irs	0.711	0.830	0.856	irs
	Heilongjiang	0.631	0.919	0.687	irs	0.716	0.930	0.771	irs
	Shanxi	0.637	0.876	0.727	irs	0.637	0.881	0.722	irs
	Neimenggu	0.495	0.801	0.618	irs	0.495	0.800	0.618	irs
	Jilin	0.487	0.773	0.630	irs	0.488	0.791	0.617	irs
Mean	0.786	0.936	0.833		0.804	0.939	0.849	
Severe aging	Shanghai	1	1	1	–	1	1	1	–
	Liaoning	0.636	0.884	0.719	irs	0.668	0.896	0.745	irs
Mean	0.818	0.942	0.860		0.834	0.948	0.873	
National Average	0.770	0.949	0.808		0.804	0.957	0.837	

drs denotes diminishing returns to scale, irs denotes increasing returns to scale, and – denotes constant returns to scale.

^a^Initial Values in Phase I: In the first stage of the three–stage DEA model, data is input into the DEA–BCC model to obtain adjusted efficiency values.

^b^Third–stage adjustment values: Within the three–stage DEA model, the effects of environmental factors and random disturbances are eliminated to derive adjusted input variable indicators. These adjusted values are then employed as new input variables, whilst the original output variables remain unchanged. The data is subsequently inputted into the DEA–BCC model to obtain adjusted efficiency values.

As shown in Table 5, the national average comprehensive efficiency of healthcare resource utilization in 2022 stood at 0.770, indicating that the overall efficiency of healthcare resource allocation in China remains suboptimal and has yet to reach its peak. Pure technical efficiency stood at 0.949, representing a relatively high level. This indicates that China's healthcare system has achieved certain successes in medical technology and management standards, though room for improvement remains. Scale efficiency was 0.808, revealing that a considerable number of regional medical institutions face challenges in realizing economies of scale. This failure to fully achieve economies of scale has significantly impacted the overall efficiency of healthcare resource utilization.

Analyzing regions with varying degrees of aging, Guangdong Province achieved a comprehensive efficiency score of 1.0 among areas experiencing mild aging. This indicates that the region's utilization of healthcare resources during this stage is at the frontier level, enabling it to fully leverage existing resources to deliver medical services. Jiangxi Province, Ningxia Hui Autonomous Region, Fujian Province, and the Tibet Autonomous Region recorded comprehensive efficiencies of 0.906, 0.765, 0.792, and 0.477 respectively. Notably, Jiangxi and Ningxia achieved pure technical efficiency of 1.0 but exhibited insufficient scale efficiency, indicating significant scope for enhancing economies of scale in these regions; Regions including Guangxi, Yunnan, Xinjiang, Guizhou, Hainan, Gansu, and Qinghai all recorded comprehensive efficiencies below 0.9, with Qinghai and Tibet exhibiting particularly low efficiency values. These areas present substantial scope for optimization in both pure technical efficiency and scale efficiency, urgently requiring enhanced management standards and adjustments to scale structures to improve healthcare resource allocation efficiency. Overall, efficiency disparities are pronounced within regions experiencing mild aging. Economically advanced provinces demonstrate relatively higher efficiency, whereas less developed western provinces face challenges in both technical and scale dimensions.

In moderately aging regions, Jiangsu and Zhejiang provinces achieved a comprehensive efficiency score of 1.0, indicating that these economically developed provinces maintain optimal utilization efficiency of healthcare resources when confronting increased medical demands arising from moderate aging. Regions including Hubei, Anhui, Henan, Sichuan, Chongqing Municipality, and Hunan recorded comprehensive efficiencies ranging from 0.822 to 0.942. Among these, Hubei, Anhui, Henan, and Sichuan achieved near- or full-scale technical efficiency of 1.0, yet exhibited insufficient scale efficiency, indicating scope for improvement in economies of scale; Tianjin Municipality, Heilongjiang Province, Shanxi Province, Inner Mongolia Autonomous Region, and Jilin Province exhibited notably low overall efficiency. These regions faced deficiencies in both pure technical and scale efficiency, particularly Inner Mongolia and Jilin, where pure technical efficiency stood at merely 0.801 and 0.773 respectively, highlighting urgent need for enhancement in medical technology and management standards. Notably, Henan, Sichuan, and Shandong exhibit diminishing returns to scale, suggesting their healthcare institutions may have become excessively large, resulting in diseconomies of scale. Efficiency improvements require optimized resource allocation and refined management practices. Overall, efficiency performance in moderately aged regions correlates closely with economic development levels. The Yangtze River Delta region demonstrates outstanding performance, while certain provinces in Northeast and North China face significant challenges.

In regions experiencing severe aging, Shanghai's comprehensive efficiency reached 1.0, indicating its healthcare resource utilization operates at the production frontier and is fully capable of addressing the medical challenges posed by advanced aging. Conversely, Liaoning Province recorded a comprehensive efficiency of 0.636, with pure technical efficiency at 0.884 and scale efficiency at 0.719, revealing relatively low efficiency in healthcare resource utilization within the region. Liaoning's lower scale efficiency suggests potential diseconomies of scale within healthcare institutions, while its suboptimal pure technical efficiency indicates room for improvement in both medical technology standards and management efficiency. This may correlate with factors such as industrial restructuring in Northeast China, high population aging rates coupled with outmigration, leading to mismatches between healthcare resource allocation and demand, as well as relatively lagging technological updates. These issues consequently constrain overall improvements in healthcare resource utilization efficiency.

Overall, the mean comprehensive efficiency does not decline with increasing aging intensity; rather, regions with mild aging exhibit the lowest efficiency, while those with severe aging demonstrate relatively higher efficiency. Concurrently, both the mean pure technical efficiency and the mean scale efficiency exhibit an upward trajectory alongside increasing aging levels. This finding indicates that the relationship between population aging and healthcare resource utilization efficiency is not straightforwardly linear. Regions experiencing severe aging (such as Shanghai) may have accumulated more mature experience in healthcare resource allocation and service process optimization through long-term adaptation to aging pressures, thereby achieving higher efficiency levels. Conversely, regions with low aging pressures, despite facing lesser demographic challenges, may exhibit lower overall efficiency due to constraints such as economic development levels, healthcare investment, and management models. This underscores that enhancing healthcare resource efficiency hinges on optimizing resource allocation structures, elevating management standards, and advancing technical capabilities to effectively address aging pressures.

#### 4.1.2 Second-stage SFA regression analysis

In the second stage, an SFA regression model was established with the relaxed variables of the input variables (calculated in the first stage) as the explained variables and the standardized environmental variables as the explanatory variables. This estimated the impact of the three environmental variables on the relaxed quantities of the input variables and subsequently eliminated them. Using Frontier 4.1 software as the computational environment, a time-varying SFA model was selected, wherein the time-varying model implies that the impact of statistical noise changes over time. It should be noted that the SFA regression in this paper was conducted independently for each year, meaning that three input slack equations were estimated separately for each year, yielding a total of 36 sets of regression results. The rationale for this approach is twofold: First, environmental factors may exhibit temporal heterogeneity, and mixed-panel regression could obscure these annual variations. Second, the adjustments required by three-stage DEA necessitate relative comparisons within the same year; conducting adjustments annually allows for more precise isolation of the impact of environmental factors specific to that year. Taking the 2022 results as an example, the estimation results are presented in Table 6.

**Table 6:** Regression results for the SFA Model of input relaxation variables with stage II environmental variables.

**Variable**	**Number of healthcare technicians per thousand population**			**Number of healthcare institution beds per thousand population**			**Number of healthcare institutions**		
	**Parameter value**	**Standard error**	**T–value**	**Parameter value**	**Standard error**	**T–value**	**Parameter value**	**Standard error**	**T–value**
Coefficient	−0.19	0.095	−1.99	−0.096	0.054	−1.770	−1082.24	459.189	−2.357
Fiscal subsidy income	0.107	0.095	1.116	−0.038	0.091	−0.417	762.785	361.486	2.110
Old–age dependency ratio	−0.05	0.041	−1.26	0.021	0.038	0.561	724.730	297.971	2.432
Per capita regional gross domestic product	0.006	0.043	0.148	0.005	0.040	0.115	−1404.01	215.419	−6.518
Urbanization rate	−0.05	0.088	−0.52	0.023	0.081	0.279			
σ^2^	1.482	0.372	3.988	0.891	0.211	4.212	58193297	1	58164481
γ	0.933	0.017	54.57	0.856	0.036	23.98	0.644	0.015	42.002
Log–likelihood	−166.059			−207.755			−3914.604		
Log–likelihood ratio test value	710.129			340.124			550.485		

As Table 6 demonstrates, the model's establishment possesses robust statistical validity and theoretical significance. The γ values for all three input slack variables are highly significant at the 1% level, confirming that the management inefficiency component dominates within the composite error term. This validates the necessity of employing SFA for error decomposition, indicating that the second-stage modelling rests upon a solid statistical foundation.

Specifically, environmental variables exert markedly divergent effects on the redundancy of different healthcare resource inputs, underscoring the necessity of incorporating environmental factors into efficiency assessment frameworks. Per capita regional GDP exerts a significant negative influence on the redundancy of healthcare institutions, indicating that heightened economic development levels help curb institutional redundancy-reflecting synergistic effects between market mechanisms and optimal resource allocation. Conversely, fiscal subsidy income and the old-age dependency ratio exert a significant positive influence on this redundancy. This reflects a potential tendency towards ‘extensive' subsidies in current fiscal investments, while accelerated aging exacerbates structural imbalances within the healthcare service system, which cannot be effectively addressed through adjustments to the number of institutions. Notably, external environmental variables exerted no significant influence on the allocation of healthcare personnel or bed resources, with their redundancy primarily driven by constant terms and random factors. This suggests that the utilization efficiency of human and material resources is more likely governed by unobserved factors within healthcare institutions, such as management standards, incentive mechanisms, and operational processes. The γ values across all three models were highly significant, strongly supporting managerial inefficiency as the primary component of input redundancy. This validates the rationale for employing the SFA model to decompose random noise and managerial inefficiency. Although certain environmental variables were insignificant in other input equations, this reflects the heterogeneity of external factor impacts across different resource dimensions rather than model specification issues.

In summary, the SFA regression results endorse the applicability of the three-stage DEA model. The significant presence of managerial inefficiency demonstrates that the raw efficiency scores from the first-stage DEA indeed confound environmental factors and random disturbances. The second-stage model successfully isolates these influences, thereby providing a basis for the third stage to derive efficiency scores reflecting genuine managerial performance. Consequently, the adoption of the three-stage DEA framework throughout this study is both appropriate and effective.

#### 4.1.3 Third stage DEA analysis

Using Excel software to adjust input variables, we eliminated the effects of environmental factors and random disturbances to obtain adjusted input variable indicators. These were then employed as new input variables while maintaining the original output variables unchanged. Substituting the data into the DEA-BCC model yielded adjusted efficiency values. Taking 2022 as an example, the results are presented in Table 5.

Comparing healthcare resource utilization efficiency before and after adjustment reveals that, after eliminating environmental variables and random disturbances, China's comprehensive efficiency mean rose from 0.770 to 0.804, pure technical efficiency increased marginally from 0.949 to 0.957, while scale efficiency improved significantly from 0.808 to 0.837. The three-stage DEA adjustment results confirm that China's healthcare resource efficiency is significantly influenced by external environmental factors, particularly concerning economies of scale. In many regions, the actual potential for scale efficiency is obscured by adverse conditions. The adjustment not only provides a more accurate reflection of management capabilities but also highlights the importance of improving scale allocation and addressing external constraints to unlock efficiency gains. This offers a clearer pathway for further optimizing healthcare resource allocation.

From the perspective of aging severity, in regions experiencing mild aging, the mean pure technical efficiency increased from 0.969 to 0.983, scale efficiency rose from 0.764 to 0.814, and the mean comprehensive efficiency improved from 0.741 to 0.800 compared to pre-adjustment levels. This indicates that after removing environmental disturbances, both the management potential constrained by external adverse factors and the economies of scale inherent in the region's overall healthcare resource utilization were effectively unleashed. while the average overall efficiency improved from 0.741 to 0.800. This indicates that the management potential and economies of scale, which were previously constrained by external adverse factors in the utilization of healthcare resources in these regions, have been released after environmental disturbances were eliminated. The dual drivers of optimizing both technical efficiency and scale allocation have collectively contributed to the improvement in overall efficiency. At the provincial level, Jiangxi, Fujian, and Guangdong maintained leading positions with both overall and pure technical efficiency at 1, indicating these regions had reached a stable and efficient production frontier in healthcare resource utilization. Conversely, Ningxia, Guangxi, Yunnan, Xinjiang, and several other provinces saw significant improvements in adjusted overall efficiency. Notably, Yunnan's efficiency surged from 0.896 to 0.980, revealing that its true resource allocation capacity had been severely underestimated by environmental factors. These findings suggest that in regions experiencing mild aging, improving external conditions and optimizing scale structures play a crucial role in unlocking healthcare efficiency potential. The scope for efficiency gains in certain western provinces warrants particular attention.

In moderately aging regions, the adjusted mean values for pure technical efficiency, scale efficiency, and overall efficiency all exhibit an upward trend. This indicates that previously, adverse external factors simultaneously masked both management potential and economies of scale within these areas. Following the removal of environmental interference, both aspects improved, jointly driving the enhancement of overall efficiency. At the regional level, Jiangsu and Zhejiang provinces maintained overall efficiency at 1 both before and after adjustment. This indicates that external environmental factors and random disturbances did not substantially impact healthcare resource utilization efficiency in these areas. When confronted with increased healthcare demand stemming from moderate aging, they still maintained high resource utilization efficiency, likely owing to their robust economic strength, comprehensive medical facilities, and advanced medical technology. Multiple regions including Hubei, Anhui, and Chongqing saw efficiency gains post-adjustment, reflecting that their genuine healthcare resource utilization capacity had previously been constrained by environmental factors. In contrast, regions such as Inner Mongolia, Jilin, and Heilongjiang exhibited persistently low efficiency post-adjustment. For instance, Inner Mongolia's technical efficiency stood at merely 0.800 and its scale efficiency at 0.618, indicating not only diseconomies of scale in healthcare resource allocation but also evident deficiencies in management and technical capabilities. This correlates closely with local geographical conditions, demographic structures, and levels of economic development, suggesting that these regions must concurrently enhance medical technology and management capacity while prioritizing the optimization of spatial distribution and scale structures for healthcare resources.

In regions experiencing severe aging, Shanghai's efficiency remained unchanged before and after adjustment. This may be attributed to its status as an economically developed region, possessing abundant healthcare resources, advanced medical technology, and a comprehensive healthcare service system, enabling it to effectively address the challenges posed by severe aging. After controlling environmental variables, Liaoning Province demonstrated improvements in pure technical efficiency, scale efficiency, and overall efficiency. This indicates that its healthcare system possesses latent potential in technological application, management capabilities, and the alignment of resource scale with service demand, which has been suppressed by external environmental constraints. This outcome indicates that Liaoning's healthcare resource utilization was previously significantly constrained by adverse external conditions such as industrial structure and population mobility. Its true management standards and scale allocation rationality surpassed initial assessments. Once these negative environmental factors are stripped away, latent efficiency is released, allowing its controllable technical, managerial, and scale allocation rationality to be more objectively reflected, resulting in a significant overall efficiency improvement.

The adjusted mean comprehensive efficiency scores for regions with varying degrees of aging were all higher than their pre-adjustment values, exhibiting a trend of increasing efficiency with deepening aging. This indicates that the healthcare demand pressures arising from aging do not necessarily lead to diminished resource utilization efficiency; rather, they may drive regions to develop more mature resource allocation models and management mechanisms in their long-term responses. The overall improvement in adjusted efficiency reflects that external environmental factors exert varying degrees of suppression across all types of regions. However, by optimizing internal management, improving scale allocation, and fostering a supportive external environment, all regions can significantly unlock latent healthcare resource efficiency. Particularly noteworthy is that regions with severe aging (such as Shanghai) maintain efficiency leadership even after adjustment. This further underscores that the key to addressing aging lies not only in resource investment but crucially in establishing efficient resource allocation systems that align with aging-related demands.

### 4.2 Dynamic efficiency analysis

#### 4.2.1 Calculation of Malmquist index results

The Malmquist productivity index was employed to calculate the total factor productivity indices and their decomposition indices for healthcare resource utilization across regions (21). Input indicators utilized values adjusted via a three-stage DEA model, representing comparable inputs within a ‘homogeneous environment'. As environmental variables' impact on outputs had been indirectly eliminated through input adjustments, forcibly adjusting outputs risked distorting measures of technical efficiency and technological progress; thus, output values were typically retained as raw data.

Using DEAP 2.1 software as the computational environment, the Malmquist indices for healthcare resources across China's 31 provinces, municipalities, and autonomous regions were calculated; the results are presented in Table 7.

**Table 7:** Malmquist productivity index and component indices for healthcare resource utilization in China's 31 provinces, municipalities and autonomous regions, 2010–2022.

**Level of aging**	**Region**	**Change in technical efficiency (EC)**	**Technological change (TC)**	**Pure technical change (PTC)**	**Scale efficiency change (SC)**	**Total factor productivity change (TFP)**
Mild aging	Fujian	0.981	0.982	1	0.981	0.963
	Gansu	0.989	0.980	1.012	0.977	0.969
	Guangdong	1	1.029	1	1.000	1.029
	Guangxi	0.989	0.972	0.997	0.992	0.962
	Guizhou	0.986	0.978	0.994	0.992	0.965
	Hainan	0.977	0.962	1	0.977	0.940
	Jiangxi	0.992	0.961	1	0.992	0.953
	Ningxia	0.987	0.961	1	0.987	0.949
	Qinghai	0.979	0.973	0.998	0.981	0.953
	Tibet	0.942	0.999	1	0.942	0.940
	Xinjiang	0.982	1.013	1.005	0.978	0.995
	Yunnan	1.002	1.010	1.003	0.999	1.012
Mean		0.984	0.985	1.001	0.983	0.969
Moderate aging	Anhui	0.997	1.012	1	0.997	1.009
	Beijing	0.983	1.001	0.994	0.989	0.984
	Hebei	0.983	0.996	0.999	0.983	0.978
	Henan	0.992	1.030	1	0.992	1.023
	Heilongjiang	0.986	1.015	1.004	0.982	1.001
	Hubei	0.996	1	0.999	0.997	0.996
	Hunan	0.997	0.991	1	0.997	0.988
	Jilin	0.970	0.999	0.999	0.970	0.968
	Jiangsu	1.003	1.052	1	1.003	1.054
	Neimenggu	0.963	0.987	1.001	0.963	0.950
	Shandong	0.995	1.063	1	0.995	1.058
	Shanxi	0.992	0.987	1.015	0.978	0.980
	Shaanxi	0.982	0.988	0.994	0.988	0.971
	Sichuan	1	1.006	1	1	1.005
	Tianjin	0.969	0.974	1	0.969	0.944
	Zhejiang	1.002	1.009	1	1.002	1.011
	Chongqing	0.996	0.998	1.004	0.992	0.994
Mean		0.989	1.006	1.001	0.988	0.995
Severe aging	Shanghai	1	0.997	1	1	0.997
	Liaoning	0.997	0.995	1.018	0.979	0.991
Mean		0.999	0.996	1.009	0.990	0.994
2010–2011		0.957	1.468	0.975	0.982	1.405
2011–2012		1.030	0.959	1.011	1.018	0.987
2012–2013		0.988	0.967	1.005	0.984	0.956
2013–2014		0.999	0.931	0.994	1.005	0.931
2014–2015		0.989	0.953	1.002	0.986	0.942
2015–2016		1.015	0.931	0.998	1.017	0.945
2016–2017		0.982	1.044	0.994	0.989	1.026
2017–2018		1.008	0.921	1.008	1	0.929
2018–2019		1.003	0.884	1.001	1.003	0.887
2019–2020		1.008	0.834	1.051	0.960	0.841
2020–2021		0.981	1.136	0.969	1.012	1.115
2021–2022		0.893	1.065	1.009	0.885	0.951
National average		0.987	0.997	1.001	0.986	0.984

Total Factor Productivity = Change in Technical Efficiency × Change in Technological Progress = (Pure Technical Efficiency × Scale Efficiency) × Technological Progress.

#### 4.2.2 Analysis of total factor productivity results

(1) Analysis of overall national medical resource utilization efficiency.

Between 2010 and 2022, the national healthcare system's average total factor productivity (TFP) stood at 0.984, indicating a slight overall decline in healthcare resource utilization efficiency during this period, with a cumulative decrease of approximately 1.6%. This decline was primarily driven by persistently insufficient technological progress (TC = 0.997), compounded by a marginal deterioration in technical efficiency (EC = 0.987).

From a dynamic perspective, healthcare productivity growth has been highly unstable and lacking in continuity. The national TFP change index reached a peak of 1.405 during 2010-2011, largely attributable to rapid technological progress (TC = 1.468) during this period. This surge may have stemmed from the concentrated release of policy dividends during the initial phase of healthcare reform and increased state investment, which significantly advanced the technological frontier of the healthcare system and elevated resource utilization efficiency. However, the TFP change index declined to 0.841 in 2019-2020, representing a relatively low level for the period. Although pure technical efficiency improved during this year (PTC = 1.051), this was offset by a significant decline in technological progress (TC = 0.834). This clearly indicates that the COVID-19 pandemic primarily suppressed the healthcare system's overall technological innovation and diffusion processes. Although internal management efficiency was forcibly enhanced under emergency conditions, it could not compensate for efficiency losses stemming from stagnation or even contraction at the technological frontier. Subsequently, during 2020-2021 and 2021–2022, the TFP change indices stood at 1.115 and 0.951 respectively, demonstrating a fluctuating recovery. The robust rebound in 2020-2021 (TFP = 1.115) was chiefly driven by the recovery in technological progress (TC = 1.136), reflecting the healthcare system's absorption of new experiences and application of novel technologies during pandemic response. However, efficiency declined again in 2021–2022, chiefly attributable to a significant deterioration in technical efficiency (EC = 0.893). This indicates that the emergency mode of technological advancement lacked sustainability, with post-pandemic healthcare systems exhibiting a degree of laxity in resource allocation and management capabilities. Overall, growth in China's healthcare productivity remains heavily reliant on reactive adjustments to external shocks, with no stable, endogenous mechanism for technological advancement and efficiency improvement yet established.

(2) Analysis of Healthcare Resource Utilization Efficiency Across Regions with Different Degrees of aging.

In regions experiencing mild aging, the mean EC value stands at 0.984. Yunnan Province (1.002) and Guangdong Province (1.000) demonstrate the highest performance, while Tibet (0.942) exhibits a relatively lower value. This indicates that most regions show limited improvement in “catching up” to the production frontier. The average TC index stands at 0.985, with all provinces below 1, confirming widespread and persistent technological regression or stagnation in this region. This may stem from chronic underinvestment in R&D and sluggish technology diffusion. The PTC mean of 1.001 and SC mean of 0.983 indicate that, under existing technological conditions, management capabilities remain largely stable while economies of scale have slightly deteriorated. Furthermore, the TFP mean for mildly aging regions stands at merely 0.969, reflecting a declining trend in overall healthcare resource utilization efficiency. Efficiency losses primarily stem from insufficient technological progress (TC < 1), whose negative impact outweighs the minor drag from technical efficiency (EC < 1). Compared to moderately aged regions (TFP mean 0.995) and severely aged regions (TFP mean 0.994), mildly aged regions exhibit the weakest productivity performance, challenging the simplistic assumption that aging inevitably suppresses efficiency. The findings suggest that in regions facing relatively lower aging pressures, the absence of urgency may conversely lead to insufficient investment and weak motivation in medical technological innovation and application, resulting in lagging productivity growth.

In moderately aged regions, the average TFP stood at 0.995, performing slightly better than in mildly aged areas, though overall efficiency still showed a slight decline. Jiangsu Province (TFP = 1.054) and Shandong Province (TFP = 1.058) achieved robust productivity growth through significant technological progress, emerging as efficiency leaders within this category. Provinces such as Henan (TFP = 1.023) and Anhui (TFP = 1.009) also enhanced efficiency through technological advancement. This indicates that some moderately aging regions successfully transformed the pressure of healthcare demands into an impetus for advancing technological frontiers. However, significant disparities exist within this category. Tianjin Municipality, Inner Mongolia, and Jilin Province exhibit low TFP values, primarily constrained by sluggish technological progress and deteriorating technical efficiency. Tianjin suffers from dual deficiencies in technical efficiency and technological advancement, resulting in severe efficiency decline. Compared to heavily aged regions, moderately aged areas demonstrate no clear overall efficiency advantage, with greater internal disparities instead. Evidently, the capacity of moderately aged regions to achieve productivity growth hinges critically on overcoming bottlenecks in technological advancement.

Within heavily aged regions, the Malmquist indices for Shanghai and Liaoning reveal marked contrasts. Shanghai's TFP change index stands at 0.997-marginally below unity-yet both its EC and PTC remain at frontier levels. Its slight productivity decline stems entirely from a minor stagnation in technological progress. This indicates that under severe aging pressures, Shanghai's healthcare system management and technological application efficiency have reached and sustained high levels, with future improvement heavily dependent on breakthrough technological innovation. In contrast, Liaoning Province's TFP change index of 0.991 similarly shows decline. The composition of this decline differs from Shanghai's: while technological progress remains insufficient, the more critical factor is a marked deterioration in scale efficiency, with pure technological efficiency gains failing to fully offset losses in the former two areas. This reflects a more complex predicament for Liaoning, characterized not only by slow advancement along the technological frontier but also by a declining match between the scale of healthcare resource allocation and the structure of aging-related demand, potentially indicating resource misallocation or diseconomies of scale.

## 5 Research conclusions and recommendations

### 5.1 Research conclusion

In the healthcare sector, the production frontier for medical services evolves over time as new medical technologies, equipment, and pharmaceuticals continually emerge. Static or dynamic analysis alone may present certain limitations. Integrating the static efficiency assessment of the three-stage DEA model with the dynamic efficiency analysis of the Malmquist index provides mutual corroboration and complementarity. This approach enables a comprehensive evaluation of China's healthcare resource utilization efficiency across both static and dynamic dimensions. It not only reveals current efficiency levels but also captures evolving efficiency trends, thereby providing a more accurate grasp of the actual circumstances surrounding healthcare resource utilization.

#### 5.1.1 The degree of aging is not a determining factor in healthcare efficiency

Analysis of regions with varying degrees of aging indicates that the impact of aging on healthcare resource utilization efficiency is neither linear nor decisive. The core explanatory variables for efficiency disparities lie in the structure of healthcare resource allocation and management effectiveness. Research indicates that regions with mild aging exhibit the lowest average comprehensive efficiency, whereas those with severe aging demonstrate higher averages. Shanghai maintains a frontier level of comprehensive efficiency at 1 despite its severe aging context. This seemingly contradictory phenomenon reveals an underlying logic: regions with higher aging levels, having long grappled with healthcare demand pressures, often drive earlier integration of medical resources and optimization of service processes, thereby developing more mature resource allocation models. For example, Shanghai has innovated its long-term care insurance system to cover home-based, community, and institutional care. This initiative guides private capital into the silver economy, cultivates a professional care workforce, and establishes a resource-sharing framework characterized by “government guidance, market operation, and social participation.” Conversely, regions with lower aging levels, despite facing lesser pressures, may suffer from inefficiency due to fragmented investment, lax management, or diseconomies of scale. The significant improvement in national scale efficiency following the three-stage DEA adjustment further corroborates the inhibitory effect of external environmental factors on scale allocation. It underscores the importance of unlocking efficiency potential through optimized management mechanisms and resource allocation structures. Consequently, the key to addressing aging challenges lies in establishing a lean management system aligned with demographic structures, rather than attributing outcomes solely to the degree of aging itself.

#### 5.1.2 Insufficient scale efficiency constitutes a key bottleneck constraining overall healthcare efficiency

Analysis of healthcare resource utilization efficiency at national and regional levels reveals that inadequate scale efficiency has become a critical structural shortfall hindering the enhancement of China's healthcare system's overall effectiveness. At the static efficiency level, the national average scale efficiency stands at merely 0.808, significantly below the pure technical efficiency of 0.949. This indicates a widespread imbalance in the alignment between resource inputs and service scale across healthcare institutions. From a dynamic perspective, the scale efficiency change index consistently remains below 1, further confirming the lag in the process of optimizing scale allocation. Notably, the issue of low scale efficiency is prevalent across regions with varying degrees of aging populations. Whether manifested as insufficient scale in underdeveloped regions due to resource dispersion (reflecting increasing returns to scale) or excessive scale in some developed regions (indicating decreasing returns to scale), both point to a systemic mismatch between resource allocation and demand structures. The marked improvement in scale efficiency following the three-stage DEA adjustment particularly demonstrates that, once the suppressing effects of external environmental factors on economies of scale are removed, regions still possess considerable potential for operating at optimal scale.

#### 5.1.3 Insufficient technological progress: the core constraint on healthcare total factor productivity growth

Dynamic efficiency analysis of the national healthcare system from 2010 to 2022 indicates that insufficient technological progress is the core factor behind sluggish or even declining total productivity growth. Malmquist index results reveal that the national average technological progress change stood at merely 0.997, remaining stagnant or even slightly declining over the long term, directly dragging down the average total factor productivity to 0.984 during the same period. This phenomenon manifests across regions with varying degrees of aging: mildly aged regions exhibit the weakest technological progress, while severely aged regions show no comparative advantage. Only a few moderately aged provinces achieved positive productivity growth through significant technological advancement. Particularly noteworthy is the sharp fluctuation and decline in technological progress indicators during external shocks (such as the COVID-19 pandemic). This reflects that China's healthcare system has yet to establish stable endogenous drivers for technological innovation and diffusion mechanisms. It remains highly reliant on policy dividends and emergency responses, making it difficult to sustain continuous productivity growth.

#### 5.1.4 Environmental factors exert a significant impact on healthcare resource utilization efficiency

Empirical analysis indicates that environmental factors exert a significant and systematic influence on healthcare resource utilization efficiency in China. The key value of the three-stage DEA model lies in its effective separation of environmental variables from random noise interference. The results clearly demonstrate that, after controlling environmental factors, the overall efficiency, pure technical efficiency, and scale efficiency of both the national level and each aging subgroup generally improved, with particularly notable gains in scale efficiency. This confirms that the external environment previously exerted an overall suppressing effect on genuine management efficiency. Specifically, heightened economic development levels help curb institutional redundancy, reflecting the optimizing role of market mechanisms. Conversely, fiscal subsidy mechanisms and aging processes may exacerbate resource misallocation due to structural and demand mismatches. The study concludes that evaluating and enhancing healthcare efficiency must account for its socio-economic context. Only by combining optimized external support conditions with improved internal management can the healthcare system's latent efficacy be fully unleashed.

### 5.2 Recommendations

#### 5.2.1 Implement a differentiated regional medical resource allocation program

Differentiated resource allocation standards shall be established based on regions categorized as experiencing mild, moderate, or severe aging, with tailored regional healthcare resource allocation schemes implemented. Firstly, regions experiencing mild aging must dynamically monitor population structures and healthcare demands to strictly control the scale of large-scale medical institution development, prioritizing the reduction of inefficient and duplicative investments. Efforts should focus on establishing efficient and accessible “15-min healthcare service zones,” strengthening community health centers' capacity to treat common and recurrent illnesses, and integrating surrounding clinics, pharmacies, and other social resources to form a service network grounded in health management. Second, targeted resource allocation must be strengthened to precisely empower high-demand regions. Addressing the relatively inadequate healthcare capacity in moderately and severely aging regions (such as Liaoning and Heilongjiang), robust mechanisms for directing resources must be established. Eastern provinces with abundant medical resources should be encouraged to forge cross-regional, institutionalized support partnerships with the more aged central, western, and northeastern regions. Through tiered, targeted interventions, this approach avoids a one-size-fits-all resource allocation while better aligning healthcare resources with demand across regions with varying degrees of aging. Ultimately, this will drive overall efficiency gains.

#### 5.2.2 Promoting digital and intelligent healthcare service models

Leveraging digitalization and intelligent technologies, actively promote digital healthcare service models such as remote consultations, telemedicine, and AI-assisted diagnosis. This approach transcends the physical limitations of traditional healthcare services, using technological dividends to offset the increasing marginal costs associated with an aging population. Firstly, vigorously develop and popularize digital services including telemedicine, online consultations, and intelligent health management. This creates a convenient online consultation channel for older adults with limited mobility, significantly reducing unnecessary trips to outpatient clinics. Combined with IoT technologies like smart wearables and home monitoring devices, continuous real-time monitoring and data feedback on vital signs and medication adherence can establish a digital defense line. This enables early warning for acute events and personalized management of chronic conditions, significantly advancing the window for intervention. Secondly, centered on contracted family doctor services, stable contractual relationships enable family doctor teams to deliver integrated care encompassing basic medical treatment, public health services, and personalized health management for contracted older adults. This service should span the entire continuum, encompassing health assessments, long-term follow-up for chronic conditions, precise medication guidance, functional rehabilitation, and necessary referral coordination. This ensures seamless, end-to-end health management for the older adults as they transition between different health statuses and healthcare institutions, fundamentally enhancing service continuity and coordination. The implementation of this model significantly enhances the convenience and accessibility of healthcare services, proving particularly significant for older adults patients in remote areas or those facing transport difficulties.

#### 5.2.3 Strive to optimize the external environment for healthcare resource utilization

Strive to optimize the external environment for healthcare resource utilization, reduce efficiency constraints unrelated to aging, enhance primary healthcare facilities, improve medical insurance policy coordination mechanisms, and balance regional economic development with public service provision. This will minimize environmental factors hindering efficiency and create more favorable conditions for the effective utilization of healthcare resources. First, correct resource misallocation during urbanization to promote service equity. Increase hardware investment in primary institutions, prioritizing equipment for common geriatric conditions and essential medicines. Require tertiary hospitals to transfer technical resources through remote consultations and specialist deployment, incorporating primary care capacity into hospital performance metrics. Second, establish one-to-one in-depth support relationships between tertiary urban hospitals and county-level hospitals, covering multiple dimensions including technical guidance, talent cultivation, and management optimization. Through these institutional arrangements, a collaborative development framework should be established where resources can flow, talent can be deployed to grassroots levels, and capabilities are enhanced simultaneously. This will ultimately ensure thatolder adults, regardless of urban or rural location, can access quality, accessible basic healthcare services.

## Data Availability

Publicly available datasets were analyzed in this study. This data can be found here: https://www.nhc.gov.cn/mohwsbwstjxxzx/tjzxtjsj/tjsj_list.shtml.

## Footnotes

1 Bed turnover rate = (Number of discharges÷Average number of open beds) × 100%

2 Population aging and Its Measurement Standards https://www.stats.gov.cn/zs/tjws/tjbz/202301/t20230101_1903949.html.
